# The Larynx the Source of the Vowel Sounds

**Published:** 1874-02

**Authors:** Thomas Brian Gunning


### AMERICAN JOURNAL

OF

### DENTAL SCIENCE.

Vol. VII. THIRD SERIES--FEBRUARY, 1874. No. 10.

### ARTICLE I.

### *The Larynx the Source of the Vowel Sounds.*—Continued.

BY THOMAS BRIAN GUNNING, NEW YORK.

The opinion is generally entertained that the larynx rises and falls with the pitch of the tone; that is, that it rises in singing from the lowest tone of the voice, *through the whole scale*, up to the highest, and falls with the tones in descending the scale. This error has no doubt arisen from the fact that, in a few of the lowest notes, the larynx falls, and in a few of the highest it rises; and the custom of using certain consonants in connection with different vowel sounds in vocal exercises has, by complicating the action of the parts, made the discovery of the truth a matter of extreme difficulty. But by singing up and down, through the scale of the voice, and using only *one* vowel sound, *without a consonant*, it will be found that there is an octave or more in the scale, in which the larynx neither falls nor rises, and that its movements down and up are all below and above that part of the scale; and this part of the scale is that in which we usually speak; and it will differ in pitch according to the voice.

Now, with the fingers and thumb upon the larynx, while sounding the *different* vowels upon any one tone within the speaking scale, whether in speaking or singing voice, it will be found that the relative positions of the hyoid bone and larynx change with *every* alteration of the vowel sound. In using the vowel sounds, a, e, i, o, u, pronounced *ah*, *a*, *ee*, *o*, *oo*, the larynx rises but little in *ah*; more in *a*, in which the hyoid bone also moves toward the chin; but in *ee* the thyroid cartilage is drawn up very close to the hyoid bone, which last is also drawn *much* nearer to the chin than in *a*; in sounding *o*, the larynx does not rise at all, but the membrane between it and the hyoid bone tightens, the bone descends some, and goes forward; in *oo*, the larynx rises much, the membrane is very tight, and the bone drawn forcibly forward. The English vowels can be formed only by different positions of the hyoid bone and larynx relatively to each other, and this is true of every possible modification of vowel sounds, and even in the whispering voice. By sounding any one consonant with the different vowels, and making allowance for the movement of the hyoid bone in carrying the tongue up, as in *k*, or *t*, it may be felt that the same relative changes are made for each particular vowel sound as without any consonant. In fact, this is so in all cases, whether initial or terminal consonants are used, and under any circumstances in which vocal tone is heard, for no tone is uttered except with some modification of vowel quality. Not only the nasals, and others, termed semi-vowels, but even the consonants except in their beginning or ending, and when initial or terminal (in which cases the contact of the parts above the epiglottis gives them distinctive characters,) except also, perhaps, in their sibilation and buzzing, are, as shown by the movemements in the hyoid bone, thyroid cartilage, and the membrane between them, vowel sounds, derived from the larynx.

In analyzing these movements, a striking resemblance in them is discovered in forming the highest and lowest vowel sounds. These are *i*, (sounded as ee in eel,) and *u*, (oo as in boot.) The difference is, that in the first, the hyoid bone and the thyroid cartilage are a little closer together, and the membrane perhaps rather tighter than in the other, while the hyoid bone is a little lower in *u*. In both, the hyoid bone is forcibly drawn toward the chin, with no perceptible difference, unless it be more forward in *u*. These vowels therefore afford the best opportunity for judging how the cavity of the mouth, and the organs within it, affect the tones of the vocal chords, and the sounds which pass the epiglottis; for the special qualities of these vowels are certainly the two extremes in pitch, not only according to Helmholtz, Merkel, Donders, and others, but as proved by Mr. Willis, whose opinions are entitled to the highest consideration. I shall therefore, in explaining the movements within the mouth and their effects, use the vowels according to the pitch of their special acoustic qualities. This will place them in the following order: *i*, *e*, *a*, *o*, *u*.

The lips, so prominently seen in speech, and which, like all the parts *within* the mouth, contribute to its perfect utterance, do not give any portion of the vowel quality to any vowel, except so far as they may be considered to do so by appropriately changing to give the vocal sound *as a whole*, a suitable outlet. In speaking the lower lip is specially active in movements appropriate to the labial consonants; but good vowels may be uttered while the lips are held wide apart, and in singing perfect vowels are heard while the lips are held far apart to give a proper quality, and outlet to the higher tones. Neither the cheeks, by their lining membrane and muscles; nor the muscles and the membrane which extend back to the centre of the pharynx, and control the size of the oral cavity, so far as proper tension can effect it, (although both, therefore, exert important influence upon each tone as a whole,) nor the tonsils which vary so much in size, contribute anything to the vowel qualities. The soft palate is important to the vowel sounds only in closing the entrance to the nose with suitable tension. The hard palate, and the gum and teeth of both jaws are important for their influence on, and their transmission of the voice as a whole; but all these are so different in different persons, and in the same individual at different times, that if the vowel sounds depended upon them for their special characters, speech could rarely be perfect, and would be at best so insecure, and so often lost, as to be of no general use. The vowel sounds may be perfectly and easily formed, even when the *rugæ* on the upper gum, extending up toward the roof of the mouth, are covered with a hard plate which presents a smooth polished surface to the tongue. The flexibility of the tongue, and its activity in speech, are so great that it is extremely difficult to define strictly what it does, and what it does not do, in articulation. It has been already shown that, after its loss, the vowels are perfectly enunciated, but the fact remains that the tongue when present, and in good condition, moves actively in the changes from one vowel sound to another, not only in speaking and whispering, but also in singing. In speaking and whispering the vowel *i* (ee,) the point of the tongue is down against the gum and inside of the lower front teeth, its centre rounding nearly up to the front of the hard palate. Now, if the finger is pushed up over the tongue, and curved suddenly down, it will be found that the tongue is pulled forward extremely at the centre of its base, but curving back on each side, while the epiglottis which is also drawn forward, leaving the aperture of the larynx wide open, is so far from the tongue that the end of the finger can go down between them. In *e* (a,) the tongue is lower in its centre, leaving a larger opening above, its base with the epiglottis being further back than in *i*, while they are also closer together. In *a* (ah,) the tongue is higher behind, its base further back, and close against the epiglottis, and both, also, nearer the back of the pharynx.

In *o*, the tongue is still higher behind, while its base with the epiglottis is so far back that the finger in examination presses the pharynx so much that it is difficult to bear. In *u* (oo,) these positions of the tongue up back, and down against the epiglottis, are so extreme that prolonged examination with the finger is unendurable. In *singing* the two higher vowels *i* and *e*, the tongue is not so close to the front of the hard palate, as in *speaking* them ; in singing the three lower vowels, the tongue is about as near to the back of the palate as in speaking and whispering. The tongue does not seem to alter its position, in singing, between high and low tones, except *in the notes above and below the speaking scale of the voice*

In these movements to form the vowels, the parts above the epiglottis are so arranged that brief explanation will clearly show that they do not act for the addition of the vowel qualities to the tones of the vocal chords, to any considerable extent, except in assisting to have this addition made below the epiglottis, or at the aperture of the larynx which it modifies. In sounding *i* (ee,) the cavity of the mouth is larger, compared with the outlet between the tongue and the front of the hard palate, than in the other vowels, whose overtones are lower. This, as shown previously, is contrary to the acoustic law which governs resonance, which is, that to exalt or strengthen acute tones, the resonant cavity must be smaller in proportion to its outlet, than in grave tones. The same inconsistency exists as to the other vowels, for the examination of the parts within the mouth, which I have before made, proves that the positions taken by the tongue, within the cavity of the mouth, during the utterance of the other vowels, make the outlet between the palate just behind the upper front teeth and the tongue below it, larger, and the cavity within, smaller, as the overtones which characterize the vowels lower in pitch. This shows that the cavity of the mouth, with the tongue and other parts within, do not act to add the vowel qualities to the tones of the vocal chords, during their passage through the mouth. The movements of the tongue, however, in assisting and complementing the epiglottis, show that it acts, in strict accordance with acoustic law, in concert with the epiglottis, to form the vowel qualities before their entrance into the cavity of the mouth, for, in the vowel *i* (ee,) the epiglottis is drawn so far forward that the resounding portion of the upper cavity of the larynx is shortened, and the aperture very widely opened, and, as the vowels, which are qualified by the re-enforcement of the graver overtones of the vocal chords, are sounded the epiglottis, by going back, and shutting down, not only increases the length of the upper cavity of the larynx, but also lessens the size of its outlet. In these movements it acts in concert with the hyoid bone and thyroid cartilage, previously explained, and it was seen that they are drawn forcibly forward in *i* (ee,) and in *u* (oo,) and these vowels include the two extremes of the vowel qualities.

By this co operation a short resonant cavity, with a large and clear outlet, is gained to re-enforce the high overtones of *i* (ee,) and a long cavity with small outlet is produced, opening nearly against the back of the pharynx to do the same for the low tone of *u* (oo.)

Prof. Tyndall's experiments with a tuning fork, held at the entrance of his mouth, are not only unreliable, but necessarily incorrect. The difficulty most apparent when the tuning fork is used, is to adjust the tongue, lips, &c., so that the cavity of the mouth will resound without their taking the true adjustment from life long habit. The vowel corresponding to the fork being known, this disposition of the vocal organs to assume the positions necessary for its production in speech becomes, in this experiment, almost if not quite unavoidable. He says that after adjusting the cavity of his mouth so that it resounds forcibly to the fork, he obtains the vowel sound without altering in the least the shape or size of his mouth. But it is a mistake to suppose that only the cavity of his mouth resounded, for the upper cavity of the larynx was in communication with it, and any resonance obtained included that of all the space down to the vocal chords. I adopted Prof. Tyndall's own method of holding the fork at the outside or entrance to the resonant cavity, but used bottles, jars, and tubes in the experiments detailed above, to show that the vowel qualities are not formed in the mouth. But these fixed cavities, reliable as they are and useful for this purpose, do not, however, meet all the acoustic points involved in speech ; nor is this done even in Mr. Willis' experiments with the reed and adjustable pipe, although they are so free from any liability to error, that his results are demonstrations. But these experiments do involve principles having important bearings upon the whole matter.

The human voice, formed by the vibrations of the vocal chords is, from its commencement in the larynx to its exit at the lips, subject to modification in and from the vocal tube. Although, as shown, the vowel sounds do not originate in the mouth, this cavity and the organs within and around it do effect them, and it is because of this influence and modification no doubt, that Mr. Bishop, Prof. Tyndall, and all who have treated of speech, have been misled as to their source. I purpose to show very briefly now how these parts exert their influence, and conform in their action to the demonstration already made that the *larynx* is the source of the vowel sonnds. This will be done without leading the reader deeply into a consideration of cataphonics, the examination of the acoustic features of speech not being the object of this paper.

Examination of the movements of the tongue in sounding the vowels in the order or pitch of their special qualities shows that in the vowel *a* (ah,) the tongue, like the thyroid cartilage, remains virtually still ; that as the vowel qualities ascend in pitch, the tongue rises toward the front, and leaves a larger cavity behind ; and that, on the contrary, as the vowel qualities descend in pitch, the tongue rises higher behind and enlarges the cavity in front. By these movements little change is made in the size of the mouth cavity ; the great alteration is, that in the vowels having the higher quality the resonant cavity of the mouth is shut in behind the tongue, while in those having the lower quality, the tongue rises behind, and the resonant cavity is in front.

The base of the tongue is seen to be close against the epiglottis in *a* (ah.) This thin fibro-cartilaginous cover to the aperture of the larynx is thus steadied, and this assistance of the tongue is also given to the epiglottis in all the sounds whose vowel qualities are of lower pitch. In those vowels, on the other hand, whose overtones are higher than in *a* (ah,) the tongue leaves the epiglottis free. By this the elasticity of the epiglottis, and its smooth inclined surfaces assist in forming the higher vowel qualities. In sounding *i* (ee,) it has been shown that the tongue while thus drawn forward at its base, also rises so abruptly that its centre is near the roof of the mouth, leaving only a very small outlet; and, as the *point* of the tongue is at the same time down behind the lower front teeth, its rounded surface is close to the projecting and mobile *rugæ*, so prominent in the palate where it curves up backward from inside the upper front teeth. By this the reflections from the uneven and mobile surfaces, restore the quality of the laryngeal sound, which would otherwise remain so effected by the large resonant cavity of the mouth, that it would not yield the pure sound of *ee* at the lips without extreme action of the parts entering into the formation of the upper cavity of the larynx. The lips are of less importance for *ee* than any other vowel. In hissing or sibilation, as heard in sounding *s*, the larynx merely rises with the hyoid bone to allow the tongue to reach the palate. It is seen that in the vowels whose qualities are low in pitch the tongue rises behind, and leaves the resonant cavity open in front. Thus in using the vowel *u* (oo,) in speech, especially in low tones, the lips are important to assist and give a proper outlet to the sound. When the lips are imperfect more change is necessary in the positions of the parts forming the upper cavity of the larynx. The change is easily detected by holding the fingers upon the front of the thyroid cartilage and sounding this vowel, first as it is usually spoken, and then with the lips widely opened.

The lips usually make a somewhat rounder outlet for *o*, and *oo*, than for *ee*. It is also probable that the back of the tongue and the pharynx which it lies so high against in *o* and *oo*, conform their outlet for these vowels to that at the lips. In whispering the same changes are made by the organs above the inferior vocal chords, as in speaking, but the expulsive power of the chest is weaker. In singing, however, the lips are frequently quite wide apart, and in singing *i* ( ee,) in addition to this, the tongue is never so near the roof of the mouth as in speaking, and yet within the medium scale of the voice the vowel sounds are well formed.

By these and former observations throughout this paper, it appears that the upper cavity of the larynx not only forms the vowel sounds in the first instance, but also continues to keep them perfect in the many different conditions and positions of the lips, tongue, and other parts above the epiglottis. This is confirmed by using any vowel sound in connection with and followed by the nasals *m* or *n*. It will then be found that the vowel sound is ennunciated perfectly, although associated with the buzzing consequent upon the vibration of the nostrils, and also affected by the peculiar resonance of the cavity itself. When the nasal *m* or *n* precedes the vowel, the soft palate frequently shuts off the nose at the commencement of the vowel sound, which is then heard through the lips only, and therefore free from any influence of the nasal cavity or the nostrils.

In the vowel sounds which pass through the nose the influence of the lips, tongue and cavity of the mouth is very limited, but since the larynx is active the sounds are easily distinguished.

It has been recently shown by Mme. Seiler that the female voice has only a few tones more than an octave in which all the vowels can be distinctly sung, and it has been long known that the vowel *u* (oo in boot,) cannot be sounded by some soprano singers on the highest notes of the voice, even where *i* ( ee,) is possible. There is, therefore, a more limited range to the vowel sounds than to the tone of the voice. This is owing, no doubt, to the fact that in the highest notes the hyoid bone is drawn so *forcibly* forward, and the membrane attached to it is made so tense, that no change can be made in its relation to the thyroid cartilage. The relative position of the hyoid bone is more forward for *oo* than *ee*, while the epiglottis is farther back in *oo*, and perhaps too near the back of the pharynx for the sound to escape. The limit of the power to form the vowel sounds appears to be reached only with that of the ability to change the form of the upper cavity of the larynx.

Up to this point the only reference made to the false vocal chords was the statement, after telling that the true chords, the originators of voice, were attached to the inside of the thyroid cartilage a little above the membrane felt as a notch in front of the neck, that "the false vocal chords are close above." The position and relation of these two pairs of vocal chords are very peculiar. The true or inferior chord passes back from the thyroid cartilage to the extreme end of the projecting point of the *arytenoid cartilage*, while the false or superior chord is attached above, on the receding front of the *arytenoid cartilage.* Between these chords, on each side of the larynx, the narrow slit, known as the ventricle, gives entrance at its anterior part to the membranous sac, situated between the superior vocal chord and the inner surface of the thyroid cartilage, and extending up frequently to its superior border. The investigation into the physiological action of the larynx in voice, aided by the laryngoscope, first used for this purpose by Garcia in 1855, and by others since then,—especially by Mme. Seiler when assisting Prof. Helmholtz in his investigation of the vowel tones and the registers of the female voice, as shown in her book, Philadelphia, 1870—have resulted in the opinion that the superior vocal chords exert little influence upon voice or speech. Researches, however, which left the movements of the upper cavity of the larynx in articulation undiscovered, would naturally fail to discover anything relating to the vowel sounds in the movements of the upper borders of the ventricles.

In view, however, of the fact that alteration of the inferior vocal chords cannot be made without affecting the chords close above, and of the peculiar activity in the upper cavity of the larynx during vowel sounds, together with the further fact that, in singing, the cavity of the mouth, and the surrounding organs are engaged in giving expression more to the vocal tones, than to the vowel sounds, I infer that the upper vocal chords, the ventricles, and their associated sacs, assist in forming the vowel qualities, at least, when the other parts of the apparatus of speech are specially active in vocal expression. It is also probable that, even in speech, the vowels having the higher qualities are in part formed by them. My own observations and experiments with the natural organs, and by other means, indicate that they are. But this subject may shortly be better understood; for a laryngoscope might be arranged with a double tube to fit into the roof of the mouth, over the tongue, so that the superior vocal chords in the vowel sound *ee* could be observed through one tube, proper light being thrown in through the other.

It is probable that in very few cases has the *whole* of the tongue been lost. If any is left in front of the epiglottis, it is to be expected that it would be more on the sides where the tongue goes further back, and where the muscles, which pass down from the styloid processes, enter it, and these muscles would therefore still remain to draw any portion of the tongue back. That some part does remain in front of the epiglottis in every case in which speech is left at all perfect seems probable. In cases where it has been taken away for punishment it is hardly possible that, unskillful as the executioners must be, they could get at the tongue very low down. In the case of Robert Rawlings the whole tongue was, it is said, removed because of cancer, by Mr. Nunneley in 1861, and the patient spoke well shortly after the operation. The Hon. Edward Twistleton reports and remarks upon this case very fully in his work, "The Tongue not Essential to Speech," London, 1873. At his request Rawlings submitted his mouth to Sir Charles Lyell, Dr. Milman, Professors Huxley, Owen and Faraday. The letters *t* and *d* could not be pronounced as, to form them, the tongue goes up to the gum just inside the upper front teeth, but the speech was on the whole good. It does not appear in this case that the epiglottis was seen by any of the gentlemen who examined the mouth. This silence in regard to so important a point indicates that some of the tongue yet covered the epiglottis. Judging from Professor Huxley's statement, enough of the tongue remained to give attachment to the muscles which draw the tongue back, and probably sufficient to pad the epiglottis, and hold it steady under the vibration of the low tones. Certainly there was none that could in the least affect the resonance of the mouth. If, in any case, the epiglottis were left entirely exposed, it is not likely that the speech would be as perfect as in Rawling's case. The movements made by the base of the tongue in the vowel sounds are merely like those of the epiglottis, which it complements. In addition to this its body and point aid in forming the consonants, except those formed by the lips, or by the lower lip and the upper teeth. Its movements in the mouth are regulated so as not to interfere with its relations to the epiglottis or alter the vowel quality, and in some sounds perhaps, it even assists in keeping them perfect in disadvantageous conditions of the mouth cavity. It is not surprising, therefore, that it should long be supposed that speech was more dependent upon the tongue than it really is: but notwithstanding the very great importance attached to it in regard to its participation in speech, the fact that the tongue is the *superior epiglottis* has never before been shown, nor, so far as I know, ever suspected. With this in view, it is seen how the epiglottis itself can be injured or lost, and a fair capability of speech remain. Intelligible speech is also possible after loss of the tongue, if the epiglottis is intact, so that the usefulness of the larynx is doubly secured.

The larynx is also protected by the projecting lower jaw. This bone, moved by muscles attached to its upper termination (in a line with the opening of the ear,) gives a base of support by its lower part) to the muscles of the hyoid bone and tongue, enabling them to act efficiently in vocalization and articulation. This control of the lower jaw at its upper extremities in man, so different from that of the lower animals, is associated with a marked peculiarity in the human hyoid bone. The ends of the hyoid bone in the dog, the sheep, the deer and other animals articulate "close," that is, directly with the upper cornua of the thyroid cartilage, and no play consequently is possible; in man on the other hand, great mobility is secured in these parts by means of an intervening flexible ligament, between the greater cornu of the hyoid bone, and the superior cornu of the thyroid cartilage, by which, change in their relative positions can be made at will.

Through these peculiarities of the jaw and hyoid bone the human larynx can be controlled and supplemented, and the wonderful faculty of speech is made possible.

